# Invasive and Non-invasive Stimulation of the Obese Human Brain

**DOI:** 10.3389/fnins.2018.00884

**Published:** 2018-11-29

**Authors:** Burkhard Pleger

**Affiliations:** ^1^Department of Neurology, BG University Clinic Bergmannsheil, Ruhr-University Bochum, Bochum, Germany; ^2^Department of Neurology, Max Planck Institute for Human Cognitive and Brain Sciences, Leipzig, Germany; ^3^IFB AdiposityDiseases, Leipzig University Medical Centre, Leipzig, Germany; ^4^BMBF nutriCARD, Center of Veterinary Public Health, University of Leipzig, Leipzig, Germany; ^5^Collaborative Research Centre 1052 “Obesity Mechanisms”, University Hospital Leipzig, Leipzig, Germany; ^6^Collaborative Research Centre 874 “Integration and Representation of Sensory Processes”, Ruhr-University Bochum, Bochum, Germany

**Keywords:** obesity, brain stimulation, transcranial direct current stimulation, repetitive transcranial stimulation, deep brain stimulation, vagal nerve stimulation

## Abstract

Accumulating evidence suggests that non-invasive and invasive brain stimulation may reduce food craving and calorie consumption rendering these techniques potential treatment options for obesity. Non-invasive transcranial direct current stimulation (tDCS) or repetitive transcranial magnet stimulation (rTMS) are used to modulate activity in superficially located executive control regions, such as the dorsolateral prefrontal cortex (DLPFC). Modulation of the DLPFC’s activity may alter executive functioning and food reward processing in interconnected dopamine-rich regions such as the striatum or orbitofrontal cortex. Modulation of reward processing can also be achieved by invasive deep brain stimulation (DBS) targeting the nucleus accumbens. Another target for DBS is the lateral hypothalamic area potentially leading to improved energy expenditure. To date, available evidence is, however, restricted to few exceptional cases of morbid obesity. The vagal nerve plays a crucial role in signaling the homeostatic demand to the brain. Invasive or non-invasive vagal nerve stimulation (VNS) is thus assumed to reduce appetite, rendering VNS another possible treatment option for obesity. Based on currently available evidence, the U.S. Food and Drug Administration recently approved VNS for the treatment of obesity. This review summarizes scientific evidence regarding these techniques’ efficacy in modulating food craving and calorie intake. It is time for large controlled clinical trials that are necessary to translate currently available research discoveries into patient care.

## Introduction

### Background and Motivation of This Review

Obesity and associated comorbidities such as cardiovascular and endocrinological diseases as well as cancer and dementia are spreading worldwide reaching a pandemic level (Dixon, 2010; Ebbert et al., 2014). Obesity’s complex etiology, it’s inter-individual variability in response to intervention as well as the rising incidence require novel therapeutic strategies (Roman et al., 2015). Intervening central nervous control systems of food choices, food reward processing, and homeostatic control through non-invasive and invasive brain stimulation may represent innovative ways in treating obesity.

In the following, I first introduce brain sites representing potential target regions for non-invasive and invasive brain stimulation. I highlight their functional role in energy homeostasis, food hedonism, food choices and obesity. Next, I introduce different non-invasive and invasive brain stimulation techniques before I summarize and discuss available evidence on their effectiveness in modulating food craving and calorie consumption in obesity.

### Bran Sites of Homeostasis and Hedonism

Homeostasis describes the evolutionary founded energy balance between ingestion and consumption. During fasting, hunger-signaling pathways trigger augmented attention to food cues and sensitivity to food-related rewarding stimuli. During consumption, the food’s taste and texture enter our awareness via the tractus solitarius in the brainstem, from which projections through the thalamus reach taste-associated neurons in the insular cortex and its frontal operculum (Rolls et al., 1988; Yaxley et al., 1988; Zatorre et al., 1992; Small et al., 1999). The increasing distension of the stomach wall releases neuropeptides (Schlogl et al., 2016), that initiate homeostatic feedback to the brain, mainly targeting homeostatic control sites such as the lateral hypothalamic area (Gibbs et al., 1973; Baskin et al., 1999; Grill and Kaplan, 2002; Minokoshi et al., 2004; Morton et al., 2006).

Eating for pleasure can elicit food consumption without hunger and beyond satiety. Hedonic brain systems, including the nucleus accumbens, the dorsal striatum, the ventral tegmental area and orbitofrontal cortex (OFC), assess the food’s rewarding properties (Wang et al., 2001; Saper et al., 2002; Stoeckel et al., 2008).

The nucleus accumbens, as part of the ventral striatum, is a dopamine rich area that subserves motivation, reward and reinforcement learning (Corbit and Balleine, 2016; Salamone et al., 2016). Hence, it has a significant role in (food) addiction (Volkow et al., 2013). It responds to food receipt if it is unexpected (Pagnoni et al., 2002), its responses to food cues predict subsequent food consumption (Lawrence et al., 2012), and it seems to encode the food’s value (Small et al., 2001, 2008).

The dorsal striatum is active during the consumption of food and its devaluation when eating beyond satiety (Small et al., 2001). Its consumption-related activity also seems to correspond to future gain in body weight (Stice et al., 2008).

The OFC merges gustatory input from the insula with cognitive information as well as incentive value from the limbic system, enabling a subjective rating of food (Berridge and Robinson, 2003; Rolls, 2005; Small et al., 2007). Studies in the late nineties, indicate that not only the posterior-medial OFC but also its caudolateral parts respond to different tastes and smells (Small et al., 1997; Zald et al., 1998). Based on these findings, both OFC regions were assigned a role in gustatory processing (Small et al., 1999). This corticolimbic process seems to be satiety-related, since neural responses to food in both OFC regions decrease with progressing consumption (Kringelbach et al., 2003; Thomas et al., 2015), resulting in diminished reward value and hence lowered motivation to eat.

### The Obese Brain

Obese individuals share certain behavioral pattern with drug addicts, such as craving, probably related to cue-associated enhanced activity in reward-associated brain regions (Tang et al., 2012; Volkow et al., 2013; Boswell and Kober, 2016). During eating, however, obese individuals, like drug addicts, present attenuated reward responses, probably promoting compensatory overconsumption (Wang et al., 2001; Stice et al., 2008; Johnson and Kenny, 2010). Dysfunctional dopamine release in the nucleus accumbens, the dorsal striatum and the medial prefrontal cortex may mediate these obesogenic reward effects (Geiger et al., 2008, 2009; Zhang et al., 2015).

Also, the OFC seems to play a role in the development of obesity. Its medio-caudal part was found to be structurally altered in relation to an increased body weight (Horstmann et al., 2011) and its right lateral part seems to represent certain aspects of food craving, such as intentions and plans to consume food, lack of control over eating, thoughts or preoccupation with food as well as guilt from cravings (Ulrich et al., 2016).

Reward-related brain systems are permanently interacting with the hypothalamus to guarantee energy homeostasis. Living in an environment of constant availability of high-caloric food, these closely linked systems can become independent and hedonism overpowers homeostasis. Individuals may consume food beyond satiety based on the interplay of food cues emerging of an obesogenic environment and personal traits, such as ‘neuroticism,’ ‘impulsivity’ and ‘sensitivity to reward’ (Gerlach et al., 2015), that induce an increased vulnerability to food-related rewarding stimuli (Davis et al., 2007; Bryant et al., 2008).

### The Neurobiology of Food Choices

The dorsolateral prefrontal cortex (DLPFC) was shown to be involved in self-control (Hare et al., 2009) and cognitive reappraisal (Kober et al., 2010). The reappraisal of food reduces the food’s desirability, and the degree to which individuals can decrease food desire seems to relate to self-control of eating in everyday life (Giuliani et al., 2013). During food reappraisal some studies showed an effect of body weight on the DLPFC’s activation strength (Hollmann et al., 2012; Kumar et al., 2016), whereas other studies did not find such a relationship (Yokum and Stice, 2013). Obesity-related alterations of the DLPFC’s functional and structural architecture suggest altered executive control processes during food choices. Alternatively, or additionally, those alteration may reflect adaptive executive control mechanisms attempting to compensate for dysfunctional reward or valuation processes (Horstmann et al., 2011; Hollmann et al., 2012).

The involvement of the DLPFC in food choices is supported by body weight related structural (Horstmann et al., 2011) and functional alterations (Hollmann et al., 2012), not only affecting the DLPFC, but also interconnected brain regions involved in reward (Yokum and Stice, 2017), valuation (Hollmann et al., 2012) and homeostatic processes (Horstmann et al., 2011), such as the insula, the medial OFC, the ventral/dorsal striatum (Avery et al., 2017), the hypothalamus, the medial prefrontal cortex, and the posterior cingulate (Harding et al., 2018). In lean individuals, changes in the homeostatic status are related to changes in neural activation strength and functional connectivity of gustatory and homeostatic brain regions, whereas in obese individuals such homeostatic changes are related to changes in activity and connectivity of gustatory and hedonic brain regions (Avery et al., 2017; Harding et al., 2018). In the fasting state, obesity, moreover, appears to be associated with stronger functional connectivity between brain areas involved in cognitive control, motivation, and reward, whereas these connections are largely unaffected by food intake in obese compared with lean individuals (Lips et al., 2014). These body weight-related alterations in neural activation strength and functional connectivity suggest that food choices in obese individuals are guided more by reward-seeking than by homeostatically relevant interoceptive information from the body (Lips et al., 2014; Avery et al., 2017; Harding et al., 2018).

## Materials and Methods

### Introducing Non-invasive and Invasive Brain Stimulation Techniques

The two most popular non-invasive brain stimulation techniques are repetitive transcranial magnetic stimulation (rTMS) and transcranial direct current stimulation (tDCS). Due to their restricted penetration depth, both techniques can be used to target superficially localized brain areas, such as the DLPFC or the frontal operculum/insular cortex. RTMS or tDCS applied to those regions may modulate activity also at interconnected distant brain sites, but these effects are not well investigated.

The stimulation of homeostatic (i.e., hypothalamus) or hedonic brain areas (i.e., ventral/dorsal striatum) requires non-invasive/invasive vagal nerve stimulation (VNS) or invasive deep brain stimulation (DBS). These techniques will be introduced after tDCS and rTMS.

### Non-invasive Transcranial Direct Current Stimulation

tDCS is a safe method to deliver subliminal tonic currents through two surface electrodes (anode and cathode) fixed to the head (Nitsche and Paulus, 2000; Brunoni et al., 2011). The current is weak (i.e., 1–2 mA), but can alter cortical excitability for minutes to hours depending on the duration and polarity of tDCS (Nitsche and Paulus, 2001; Hummel et al., 2005). During its application, tDCS seems to provoke a shift in membrane potentials of stimulated cortical assemblies. This is followed by modifications in the efficiency of synaptic transmission during the first 20 min after application (Clark et al., 2011; Rahman et al., 2013).

Delivered to the primary motor cortex, TDCS modifies cortical excitability in a polarity-specific manner (Stagg and Nitsche, 2011). Anodal tDCS attenuates intracortical inhibition and increases facilitation after the stimulation session, not during it. Cathodal tDCS, in turn, attenuates facilitation during the stimulation session and increases inhibition after it. On the synaptic level, tDCS is thought to modulate the excitability of cortical neurons in the cerebral motor cortex through the modulation of glutamatergic action (Stagg and Nitsche, 2011).

For sham tDCS, the current is temporarily ramped up and then ramped down again, avoiding longer active brain stimulation. With this procedure, participants experience the same tingling sensations that occur during the first few minutes of real tDCS (Gandiga et al., 2006).

### Non-invasive Repetitive Transcranial Magnetic Stimulation

The TMS coil that contains a magnetic field generator is fixed to the head over the brain site of interest. The magnetic field elicits a small electric current in the targeted cortical assemblies via electromagnetic induction. Low frequency rTMS (e.g., 1 Hz) delivered to the primary motor cortex attenuates cortical excitability without affecting cortical inhibition. High frequency rTMS (≤5 Hz), in turn, leads to an attenuation of cortical inhibition (Fitzgerald et al., 2006). Theta-burst TMS (50 Hz, TBS) can be delivered continuously (cTBS) or intermittedly (iTBS) to either inhibit or facilitate cortical excitability, respectively (Di Lazzaro et al., 2005).

During motor cortex stimulation, the TMS effect can be easily captured via simultaneously recorded motor evoked potentials of the muscle whose representation falls into the stimulated cortical motor representation. The navigation of the TMS coil over brain regions without such a direct output, e.g., the DLPFC, is more challenging and may be guided by neuro-navigation based on structural and/or functional CT/MRI of the participant’s brain.

### Non-invasive and Invasive Vagal Nerve Stimulation

The vagal nerve is a peripheral nerve that belongs to the autonomic nervous system. It is linked to parasympathetic control of the heart, lungs, and digestive tract. It also has a sympathetic function via peripheral chemoreceptors. For clinical purposes, stimulation of the vagal nerve is used to treat drug-refractory epilepsy and treatment-refractory depression (Panebianco et al., 2015; Carreno and Frazer, 2017; Edwards et al., 2017).

The vagal nerve receives afferent inputs from the stomach and is involved in signaling the homeostatic feedback to the brain. Its invasive or non-invasive stimulation is thought to reduce appetite. Non-invasive VNS for such purposes involves transcutaneous stimulation of the T6 dermatome, and hence of afferent projections to the vagal nerve.

For invasive treatment of obesity, the surgeon implants a helical coil at the abdominal trunk of the nerve proximal to the stomach. The small electric generator is implanted subcutaneously below the clavicle (Edwards et al., 2017). The stimulation is generally directed to the left vagal nerve since stimulation of the right vagal nerve could cause serious cardiologic side effects, due to the right vagal nerve’s implementation in various cardiac functions (Carreno and Frazer, 2017).

### Invasive Deep Brain Stimulation

Deep brain stimulation demands the stereotactic implantation of electrodes in deep brain structures, whereas the pulse generator is implanted subcutaneously (Khan and Henderson, 2013). Delivery of a direct electrical current by DBS can modulate the activity of dysfunctional brain circuits (Karas et al., 2013). DBS was first used as a treatment option in drug-refractory Parkinson patients. Recently, it was successfully tested as a treatment option for therapy-resistant depression, obsessive compulsive disorder and substance abuse (Karas et al., 2013; Coenen et al., 2015).

Electrode implantation is done under local or generalized anesthesia, with or without electrophysiological recordings, and with or without perioperative psychological and neurological tests (Lefaucheur et al., 2008; Xie et al., 2010; Chen et al., 2011; Foltynie et al., 2011). DBS is a relatively safe method with a mortality rate below 0.3% (Voges et al., 2006; McGovern et al., 2013). Individuals with obesity, however, had slightly higher risk of postoperative complications (Hu et al., 2017).

For the treatment of obesity, DBS electrodes are implanted to the lateral hypothalamus area or the nucleus accumbens to modify homeostatic or hedonic food responses, respectively. So far, available data suggest that the stimulation of the lateral hypothalamic area could increase resting energy expenditure, while stimulation of the nucleus accumbens may reduce food intake (Ho et al., 2015b). The mechanism of action, however, remain to be elucidated.

## Results

### Effects of Non-invasive tDCS

tDCS can be used to target superficially located brain regions, such as the DLPFC – the preferred target for non-invasive brain stimulation in obesity research. In most studies, one stimulation electrode is fixed to the right forehead (e.g., anode), while the other electrode to the left forehead (e.g., cathode). For the inverse polarity anode and cathode are simply reversed.

Previous studies accumulated evidence of an obesity-associated prefrontal imbalance (Carnell et al., 2012; Brooks et al., 2013; Vainik et al., 2013) with dysregulated DLPFC activity either in the right (Alonso-Alonso and Pascual-Leone, 2007) or the left hemisphere (Gluck et al., 2015). These alterations may point to general deficits in decision-making also supporting reduced self-reflection on food choices. The idea behind tDCS is to modulate DLPFC activity and hence executive control over everyday food choices (see Supplementary Table S1 for an overview on tDCS studies).

Based on these assumptions, a number of studies investigating the effect of a single session of bilateral tDCS to the DLPFC. Accumulating evidence suggest that a single session of tDCS delivered to the DLPFC in lean individuals reduces food craving immediate to tDCS (Fregni et al., 2008; Goldman et al., 2011; Montenegro et al., 2012; Kekic et al., 2014).

Fregni et al. (2008) showed, in a young human female sample, that food, or food-related movies, after sham tDCS increased food craving as expected. After anodal tDCS was delivered to the left DLPFC (i.e., cathodal tDCS applied to the right DLPFC), food stimuli no longer increased food craving. Moreover, for the inverse polarity (anodal tDCS applied to the right DLPFC/cathodal tDCS delivered to the left DLPFC) individuals fixated food-related pictures less frequently. Interestingly, a single tDCS session was also enough to reduce the amount of consumed food (Fregni et al., 2008).

Goldman et al. (2011) applied anodal tDCS to the right DLPFC in a young mixed-gender, overweight to obese sample while cathodal tDCS was delivered to the left DLPFC. Effects due to the inverse polarity were not tested. The ability to resist sweet foods and carbohydrates was better for real tDCS than for sham (Goldman et al., 2011).

Kekic et al. (2014) used the same electrode arrangement as Goldman et al. (2011) in a young, lean to overweight human female sample and, in line with their findings, craving for sweet, but not savory food, was less pronounced after real tDCS. Interestingly, women showing reflective choice behavior presented larger tDCS-induced anti-craving effects than those showing impulsive choice behavior (Kekic et al., 2014).

Montenegro et al. (2012) investigated the effect of a single tDCS session in addition to physical exercise in a small, mixed-gender and overweight sample. The comparison of their findings to other studies mentioned above is difficult (Goldman et al., 2011; Kekic et al., 2014), since Montenegro et al. used a slightly different arrangement of tDCS electrodes. For anodal tDCS to the left DLPFC, they attached the anode to the frontal F3 location, in correspondence with the international 10–20 EEG system. The cathode was attached to Fp2, the contralateral supraorbital area. TDCS with this electrode arrangement and in combination with physical exercise had a stronger suppressive influence on the desire to eat as compared to either tDCS or exercise alone (Montenegro et al., 2012).

Most pilot studies to date investigated the effect of a single tDCS session in small populations limiting their suitability to derive hypotheses for larger clinical trials with repetitive tDCS. To this end, tDCS’s influences on the DLPFC, associated cognitive effects as well as biochemical action mechanism must be further deciphered.

Regarding tDCS’s biochemical action mechanisms in the rat brain, Surowka et al. (2018) recently investigated molecular and elemental tDCS effects in brain regions involved in appetite control. Rats fed high-caloric nutrients while receiving prefrontal stimulation showed significantly inhibited appetite. Both, anodal and cathodal tDCS elicited qualitative and structural properties of lipids. Anodal tDCS, however, produced a larger effect on protein secondary structure. Both polarities also reduced surface masses of several electrolytes, but again anodal tDCS had a stronger effect than cathodal tDCS (Surowka et al., 2018).

tDCS studies using either single session or repetitive sessions, support the assumption of a prefrontal imbalance, however not only in obesity (Carnell et al., 2012; Brooks et al., 2013; Vainik et al., 2013), but also in lean individuals (Fregni et al., 2008; Goldman et al., 2011; Montenegro et al., 2012; Kekic et al., 2014). A recent EEG study in obese individuals revealed another imbalance, not between both DLPFCs, but between the left DLPFC and the right frontal operculum (Kumar et al., 2016). Allowing the desire for visually presented food increased activity in the left DLPFC. During regulating the desire for the same food, activity in the right frontal operculum increased (Kumar et al., 2016).

Based on this evidence, Grundeis et al. (2017) applied cathodal tDCS to downregulate activity in the left DLPFC in a young female obese sample, while simultaneously applying anodal tDCS to upregulate activity in the right frontal operculum. They assumed a tDCS-induced strengthening of the ability to regulate food desire and hence reduced calorie consumption. TDCS, however, did not modify food desire and had no influence on calorie consumption, suggesting that tDCS also had no effect on both target regions (Grundeis et al., 2017).

Contrarily to these findings, tDCS applied to both DLPFCs showed an effect on food craving and calorie consumption (Fregni et al., 2008; Goldman et al., 2011; Kekic et al., 2014; Lapenta et al., 2014). This effect might be based on feasible alterations of activity within both DLPFCs together, or of reward responses from connected dopaminergic regions such as the striatum or the OFC (Geiger et al., 2008, 2009). A direct modification of dopaminergic responses from reward regions is, however, unlikely, since tDCS lacks in necessary penetration depth.

Only few studies to date probed repetitive application of tDCS sessions on food craving and/or food consumption. Gluck et al. showed in a small, solely obese, mixed-gender human cohort that repetitive applications of anodal as compared to cathodal tDCS delivered to the right DLPFC resulted in reduced calorie consumption and greater weight loss (Gluck et al., 2015).

More recently, Ljubisavljevic et al. (2016) showed in a young, mixed-gender, lean to overweight human cohort that 5 days of anodal tDCS to the right DLPFC reduced food craving of high-caloric food, but not for carbohydrates. Interestingly, this immediate effect was followed by an after effect since both current and habitual craving were still reduced 30 days after active tDCS – an effect that was not found following a single tDCS session (Ljubisavljevic et al., 2016).

Also, Jauch-Chara et al. (2014) applied anodal tDCS to the right DLPFC in a young, solely male, lean to overweight human sample over 1 week and found a reduction of appetite resulting in reduced calorie intake by 14%. These effects on consumption of high-caloric food were recently corroborated in rats receiving tDCS over 8 days (Macedo et al., 2016).

More pilot studies with repetitive tDCS sessions, like the one by Jauch-Chara et al. (2014), Gluck et al. (2015), or Ljubisavljevic et al. (2016), are required to assess the effect and safety of repetitive tDCS sessions on eating behavior.

### Effects of Non-invasive rTMS

Like tDCS, rTMS is limited by its penetration depth, allowing the stimulation only of superficially located brain areas, such as the DLPFC. Stimulating the DLPFC with rTMS, however, elicits changes not only of the DLPFC’s activity but also of the muscles directly underneath the TMS coil. These contractions can be awkward, and they are difficult to mimic during sham stimulation.

The advantage of rTMS is that it requires only a single stimulation coil and not two electrodes like tDCS. This allows to target brain regions with higher spatial accuracy. RTMS at high frequencies has the potential to facilitate activity in the targeted DLPFC, while simultaneously inhibiting activity in connected regions such as the OFC and the anterior cingulate cortex (Nahas et al., 2001).

Previous studies suggest that rTMS may have a treatment effect in addiction and possibly also in obesity, if obesity is associated with symptoms of food addiction (Mishra et al., 2011). Addiction and food addiction share several symptoms (Gearhardt et al., 2011a, 2014), such as reward dysfunction, craving, emotion dysregulation, and impulsivity (Schulte et al., 2016). Food addicts show compulsive consumption of palatable food as well as psychological dependence expressed as craving together with heightened pleasure and excitement (Gold et al., 2004). Food addiction has a significantly greater co-morbidity with Binge Eating Disorder, depression, and attention-deficit/hyperactivity disorder compared to age- and weight-equivalent controls (Davis et al., 2011; Gearhardt et al., 2012). Food addicts present higher levels of negative affect, eating disorder psychopathology, and lower self-esteem (Gearhardt et al., 2013). They display enhanced attentional aspects of impulsivity (Meule et al., 2012; Murphy et al., 2014), and greater emotional reactivity than their obese counterparts (Davis et al., 2011; Pivarunas and Conner, 2015). In the context of food, they present greater cravings and the tendency to ‘self-soothe’ with food. Together, these observations suggest clinically relevant subtypes of obesity that may possess different vulnerabilities to environmental risk factors (Davis et al., 2011).

Brain imaging in food addicts revealed enhanced activation in the anterior cingulate cortex, medial OFC, and amygdala in response to anticipated receipt of food (Gearhardt et al., 2011b). Higher versus lower food addiction scores seems to relate to enhanced activation in the DLPFC and the dorsal striatum in response to anticipated receipt of food but less activation in the lateral OFC in response to receipt of food. These findings suggest similar patterns of neural activation in addictive-like eating behavior and substance dependence: elevated activation in reward circuitry in response to food cues and reduced activation of inhibitory regions in response to food intake (Gearhardt et al., 2011b).

Accumulating evidence suggests that rTMS influences food craving and consumption even in obese individuals who do not suffer from food addiction (see Supplementary Table S1). In young, lean to obese women with food craving, 10-Hz rTMS, a classical form of high-frequency rTMS, delivered to the left DLPFC abolished those cravings only if real rTMS was applied. After sham rTMS, cravings continuously increased over the time of food picture presentation. Consumption of food, however, did not change after real rTMS (Uher et al., 2005).

Although these findings agree with studies that investigated the effects of a single tDCS session (see “Effects of non-invasive tDCS”), they disagree with other rTMS findings again obtained from a small human cohort consisting solely of lean-to-obese women (Barth et al., 2011): In the study by Barth et al. (2011) rTMS was also delivered to the left DLPFC, but unlike the former study, real and sham rTMS were not compared between but within subjects. Food cravings significantly dropped, however, not only for real but also for sham rTMS, suggesting that real rTMS did not modulate food craving (Barth et al., 2011). A major shortcoming of this study, however, was the heterogeneity of study participants. Barth et al. (2011) mixed obese and lean women. This mixed sample may have lowered the sensitivity to detect effects on food craving in obesity. Another possible explanation is that rTMS was delivered at 100% of the motor threshold. Other studies instead have used 110%.

Reduced food craving, nevertheless, seems to represent the best reproduced DLPFC-rTMS effect in obese individuals. Previous studies have shown that this effect can last for up to 2 weeks (Fregni and Pascual-Leone, 2007).

Kim et al. (2018) were the first to show that 10Hz rTMS delivered to the left DLPFC in a mixed-gender, obese sample even reduced food consumption and hence body weight in obese individuals. They, however, applied not just one but four rTMS sessions also leading to higher levels of satiety than in the sham group. CT-guided assessments of fat distribution showed the parallel reduction of visceral adipose tissue without an excessive reduction of skeletal muscle mass. Furthermore, fasting insulin concentration and insulin resistance decreased only in the real rTMS group. Contrarily, C-reactive protein and lipid profiles remained almost unaffected probably due to the relatively short duration of the study. Together, these findings suggest that multiple rTMS sessions, like multiple tDCS sessions, have the potential to reduce body weight and may even decrease the risk for vascular events (Kim et al., 2018). Applying just a single 10Hz rTMS session seems less effective. It seems to provoke reduced food craving, but this effect seems not to translate into reduced food consumption (Uher et al., 2005).

Accumulating evidence from above-mentioned studies suggest that facilitatory high-frequency (i.e., 10 Hz) rTMS applied to the left DLPFC reduces food cravings as well as food consumption if individuals receive multiple rTMS sessions. This assumption would be indirectly strengthened, if inhibitory rTMS provokes the inverse effect. One study recently addressed this question. Lowe et al. (2018) applied inhibitory cTBS (i.e., continuous 50 Hz rTMS), targeting the left DLPFC, in a female lean to obese human sample and assessed its influences on food consumption. Only after real cTBS, women selectively consumed more high-calorie food, but not low-calorie food. With electroencephalography (EEG), Lowe et al. observed a parallel attenuation of activity in the stimulated left DLPFC region (Lowe et al., 2018). These findings support the assumption that rTMS delivered to the DLPFC can modulate food consumption in both directions.

### Effects of Non-invasive and Invasive VNS

The vagal nerve sends afferent signals from the stomach to homeostatic brain sites. Its non-invasive and invasive stimulation is therefore assumed to reduce appetite and body weight. Based on these assumptions, non-invasive VNS was supposed as a treatment option for obesity (Page et al., 2012). First findings indeed suggest a reduction in appetite and body weight (Ruiz-Tovar et al., 2014; Ruiz-Tovar and Llavero, 2016) (see Supplementary Table S1).

In a well-controlled proof-of-concept study, Ruiz-Tovar et al. (2014) found in 45 mainly female, obese individuals that diet in combination with non-invasive VNS delivered to the T6 dermatome led to reductions of body weight and appetite. In a follow-up study they also evaluated the long-term effect of non-invasive VNS. Nine months of treatment in 150 obese individuals led to a reduction in appetite associated with a mean weight loss of 14.5 (±2.8) kg (Ruiz-Tovar and Llavero, 2016).

In agreement with these positive effects of non-invasive VNS, also invasive VNS has shown promising results in human studies. Findings of the so-called ReCharge trial suggest positive effects on appetite and body weight control (Ikramuddin et al., 2014; Shikora et al., 2015). The ReCharge trial is a randomized, double-blind, sham-controlled clinical study involving 239 obese to morbidly obese, mainly female individuals (Ikramuddin et al., 2014; Shikora et al., 2015). After 12 months of non-invasive VNS, 52% of participants in the real VNS group lost 20% or more of their body weight. Thirty-eight percent at least lost 25% or more of their body weight. In the sham group, only 32% of participants lost 20% or more and 23% lost 25% or more of their body weight. Serious adverse event occurred only in 3.7% of participants (Ikramuddin et al., 2014). At 18 months, 88% of VNS individuals and 83% sham individuals remained in the study. In those participants, the weight loss was 23% in the VNS group vs. 10% in the sham group. VNS individuals largely maintained 12-month weight loss of 26%. Individuals in the sham group contrarily regained over 40% of their initial 17% weight loss (Shikora et al., 2015).

These findings were corroborated by the VBLOC DM2 study. Intermittent invasive VNS among 28 obese individuals with type 2 diabetes mellitus over 2 years proved to be a safe procedure leading not only to weight loss but also to reductions of metabolic and vascular risk factors (Shikora et al., 2016).

### Effects of Invasive DBS

There are two potential targets for DBS. One target is the lateral hypothalamus as the brain’s main homeostatic control site. The other one is the nucleus accumbens as a region of the hedonic brain circuitry underpinning motivation, reward and reinforcement learning. Converging evidence in rodents suggest that the shell and core of the nucleus accumbens are mediating reward-driven behaviors (Avena and Bocarsly, 2012; Richard et al., 2013; Burton et al., 2015). DBS of both subregions proved effective to modify such behaviors (Hamani and Temel, 2012; Luigjes et al., 2012; Muller et al., 2013; Pierce and Vassoler, 2013).

Only few studies have investigated DBS effects in the obese human brain (see Supplementary Table S1). Harat et al. (2016) reported about a 19 years old woman who developed morbid obesity after surgical extraction of a brain tumor with an intervention-induced damage to the hypothalamic area. DBS was implanted bilaterally over the nucleus accumbens. After 14 months, the women reported less appetite and food craving. This was associated with reduced body weight (Harat et al., 2016).

Deep brain stimulation has also been applied to the lateral hypothalamic area to modulate homeostatic processing (Ho et al., 2015a,b). After 35 months, bilateral DBS over the lateral hypothalamic area in three treatment-refractory patients, two females and one male, with morbid obesity led to weight loss in two patients, whereas the remaining patient at least maintained body weight (Whiting et al., 2013).

## Discussion

### Non-invasive Transcranial Direct Current Stimulation (tDCS) and Repetitive Transcranial Magnet Stimulation (rTMS)

Accumulating evidence suggests that non-invasive brain stimulation delivered to the DLPFC, a brain region assumed to underpin executive functions, may improve inhibitory control capacities (Lapenta et al., 2014; Lowe et al., 2014) over automatic processes involved in food craving (Val-Laillet et al., 2015), reward valuation processes (Camus et al., 2009) and attentional biases toward high-caloric food (Fregni et al., 2008).

RTMS (Uher et al., 2005; Barth et al., 2011) or tDCS (Goldman et al., 2011; Montenegro et al., 2012; Kekic et al., 2014; Lapenta et al., 2014) delivered in a single session to the DLPFC, appear effective to reduce food craving rather than calorie consumption (see Supplementary Table S1). Reduced food consumption (Kim et al., 2018), mainly of high-caloric food (Jauch-Chara et al., 2014), as well as sustained reductions in food craving (Ljubisavljevic et al., 2016) were achieved with multiple DLPFC stimulation sessions. TDCS was also tested in combination with standard weight-loss approaches such as aerobic exercise. As compared to tDCS alone. This combination had a stronger influence on the desire to eat (Montenegro et al., 2012). Contrarily, Grundeis et al. found no effect of tDCS on the desire for food or food consumption (Grundeis et al., 2017). They, however, attached tDCS electrodes not to the DLPFC, like in the studies mentioned above, but to the left DLPFC and the right frontal operculum (Kumar et al., 2016).

Several recent meta-analyses support evidence arising from single studies. Enhancing excitability in the DLPFC, independently of the stimulation technique, attenuates food craving and consumption of high calorie food (Jansen et al., 2013; Hall et al., 2017; Lowe et al., 2017). Based on 17 studies, Jansen et al. revealed a medium effect size for the influence of real non-invasive stimulation as compared to sham stimulation on food craving. There was no difference between rTMS and tDCS, or between left and right DLPFC stimulation (Jansen et al., 2013). Also, Lowe et al. revealed a moderate-sized effect on food cravings across 16 studies. This effect was statistically significant only for rTMS and not for tDCS. Contrarily to Jansen et al. (2013) there was not enough evidence to support a causal effect of non-invasive brain stimulation on food consumption (Lowe et al., 2017). Due to criticism regarding their initial statistical model, they revised respective analyses and in fact found a significant effect also on food consumption that was larger for left as compared to right DLPFC stimulation (Hall et al., 2017).

As for tDCS, also the influence of rTMS on the DLPFC is not fully understood. Besides a direct influence on executive functions embedded in the DLPFC, evidence for the indirect modulation of reward regions is stronger for rTMS than for tDCS. Dopamine release from the ventral tegmental area seems to be modulated by glutamatergic projections from the prefrontal cortex and glutamate receptors in the ventral tegmental area seem to trigger dopamine release in the nucleus accumbens (Taber et al., 1995). In line with these findings, rTMS targeting the DLPFC can trigger the release of dopamine not only in cortical structures, such as the anterior cingulate cortex and OFC (Cho and Strafella, 2009), but also in interconnected subcortical reward regions, such as the striatum (Strafella et al., 2001; Keck et al., 2002; Pogarell et al., 2006; Ko et al., 2008; Ahn et al., 2013), substantia nigra, and ventral tegmental area (Keck et al., 2002). This effect seems to be stronger after stimulating the left DLPFC (Ko et al., 2008). The potential remote influence of rTMS and tDCS on dopamine release in the reward circuitry as well as a direct modulatory influence on executive control via the DLPFC may account for their efficiency in reducing food craving and, if applied with multiple sessions, food consumption. Another possible mechanism of rTMS is the reduction of the brain-derived neurotrophic factor (BDNF). Since low BDNF is related to obesity (Araki et al., 2014), rTMS may reduce food consumption through increasing BDNF. Muller et al. (2000) for instance, showed that rTMS can increase BDNF mRNA expression as well as cholecystokinin in the rat brain.

The degree to which rTMS/tDCS effects on food craving and consumption are moderated by stimulation frequency (rTMS) or electric field strength (tDCS) remains unclear since most studies applied rTMS with potentially faciliatory protocols, such as 10 Hz, and tDCS with standard electric field strength, such as 2 mA over 20 min (see Supplementary Table S1). Comparing single rTMS/tDCS session studies with multi-session studies, however, suggests that multi-session protocols are more effective (Jansen et al., 2013; Hall et al., 2017; Lowe et al., 2017). Most single session studies show an effect of rTMS/tDCS only on food craving. This effect seems to translate into reduced food consumption if multiple rTMS/tDCS sessions are applied, suggesting possible associations between effect size and stimulation load (pulse number and density) (see Supplementary Table S1).

The early and long-term changes taking place after a rTMS or tDCS session seem to depend on a complex scenario of different mechanisms, including gene activation/regulation, de novo protein expression, morphological changes, changes in intrinsic firing properties, modified network properties, homeostatic processes, glial function, as well as long-term potentiation (LTP) and long-term depression (LTD) (Cirillo et al., 2017). LTP is thought to result from faciliatory stimulation protocols, such as 10 Hz rTMS, whereas LTD seems to result from inhibitory protocols, such as cTBS. A recently published study (Wiegert et al., 2018) showed that optogenetically induced LTP in rat hippocampus enhances synaptic stability over days, whereas long-term depression (LTD) destabilizes synapses. Most potentiated synapses are resistant to depression suggesting that synaptic transmission strength depends on the sequence of plasticity-inducing stimulations (Wiegert et al., 2018). High stimulation load induced by multiple rTMS/tDCS sessions may enhance the stabilizing effect on synaptic transmission in stimulated cortical areas, such as the DLPFC, with potentially larger effects on corresponding behavior. Although both, rTMS and tDCS, seem effective to reduce food craving and consumption, their methodological comparison is hampered by each technique’s distinct advantages and disadvantages. The advantage of one technique is, in most cases, the disadvantage of the other one. The advantage of rTMS is its higher spatial accuracy (Di Lazzaro et al., 2005; Fitzgerald et al., 2006). TMS coils are available in different shapes and sizes. Most studies use either round coils or figure-of-eight shaped coils. Figure-of-eight coils are spatially more specific. They produce a focal magnet field that allows to target single brain sites (Di Lazzaro et al., 2005; Fitzgerald et al., 2006). Modern rTMS systems can also be equipped with neuro-navigation. Such systems allow to integrate individual CT or MRI scans to navigate the TMS coil over the brain site of interest. TDCS instead requires anode and cathode that are both attached to the skull to initiate a current (Nitsche and Paulus, 2000; Brunoni et al., 2011). This means that tDCS does not allow to target single brain areas like rTMS. In most studies, at least two brain sites are simultaneously stimulated, one anodally and the other cathodally.

In most prefrontal tDCS studies aiming at modulating food craving and consumption, one electrode is attached to the left forebrain and the other one to the right forebrain (Goldman et al., 2011; Montenegro et al., 2012; Kekic et al., 2014; Lapenta et al., 2014). That means that both prefrontal cortices, including the DLPFCs, are simultaneously stimulated, with inversed polarity, and the current which is send between electrodes may on its way even modulate activity in the medial prefrontal valuation system. This may additionally account for the observed effects. Mapping tDCS effects on the underlying functional neuroanatomy is therefore not as straight forward as for rTMS. RTMS allows a more locally specific stimulation of the brain. Hence, observed effects are far easier to map on the underlying brain structure (Di Lazzaro et al., 2005; Fitzgerald et al., 2006).

tDCS, in turn, evokes far less side effects (Nitsche and Paulus, 2000; Brunoni et al., 2011). Especially contractions of facial muscles that usually appear during rTMS of the DLPFC are difficult to mimic during sham stimulation. Modern sham coils do not only produce the same sounds as real coils, they are also equipped with an electric device that only stimulates the underlying skin area and muscle groups, but not the brain. Stimulation induced sensations are nevertheless slightly different as compared to real TMS and therefore in principle assessable. TDCS induced side effects are far easier to mimic. The electric current provokes a tingling sensation on the skull – a sensation that in most individuals disappears shortly after the current is switched on. Afterward, these participants do not feel any stimulation-induced sensations. For sham tDCS, the current is therefore switched on like for real tDCS and then, without notice, switched off after the initial stimulation period. In some individuals, however, tingling sensations on the skull sustain the whole tDCS session. In a within-subject design, these individuals are aware of when they received real or sham stimulation.

In face of possible therapeutic applications, tDCS bears a major advantage. It consists only of a battery and two (or more) electrodes (Nitsche and Paulus, 2000; Brunoni et al., 2011). TMS devices instead are expensive and therefore restricted to labs or clinics. TDCS devices are easy to construct and affordable offering broad applicability even at home or at work. Taking together, rTMS is spatially more precise and therefore more meaningful from a scientific perspective. TDCS is easier to handle and far more affordable offering its easier translation into therapeutic use (Nitsche and Paulus, 2000; Brunoni et al., 2011).

The main limitation in the field of non-invasive DLPFC stimulation is the low number of randomized controlled studies, far more single session studies than multiple-session studies, a gender and age bias toward young females, and the lack of replication across stimulation techniques (see Table 1). Before these techniques can be applied in clinical practice, their therapeutic potential and biochemical action mechanisms must be more intensively investigated.

**Table 1:** Limitations of currently available tDCS and rTMS studies.

Few randomized studies
More single session than multiple-session studies
Gender and age bias toward young females
Lack of replication across stimulation techniques

To assess their therapeutic potential, large clinical studies are required, like a recent study on depression that compared the effect of prefrontal tDCS plus antidepressant drugs versus antidepressant drugs alone (Brunoni et al., 2017). To study tDCS’s (or rTMS’s) effects on eating behavior in obesity, corresponding clinical trials, for instance, could compare the effect of dieting to dieting plus repetitive prefrontal tDCS (or rTMS).

### Invasive Deep Brain Stimulation

As compared to tDCS and rTMS, far less evidence is available for the influence of DBS and VNS on food craving and calorie consumption (see Supplementary Table S1). For DBS, few case studies suggest that invasive stimulation of the nucleus accumbens or the lateral hypothalamic area may represent an alternative therapy for therapy-refractory morbid obesity (Whiting et al., 2013; Harat et al., 2016). In this context, DBS could in future develop as an alternative to bariatric surgery, but studies comparing their effectiveness as well as their specific advantages and disadvantages are required to assess which individuals have the largest benefit from the one or the other method. Like bariatric surgery, DBS is invasive, expensive and hence not suitable for most morbidly or non-morbidly obese individuals. Less invasive, and cheaper techniques, such as rTMS, tDCS and VNS are safer and hence better alternatives.

### Invasive and Non-invasive Vagal Nerve Stimulation

For VNS, few blinded and controlled clinical trials suggest that non-invasive (Ruiz-Tovar et al., 2014; Ruiz-Tovar and Llavero, 2016) as well as invasive VNS (Ikramuddin et al., 2014; Shikora et al., 2015, 2016) leads to sustained weight loss and glycemic control with a well-tolerated risk profile. Based on this evidence, the U.S. Food and Drug Administration recently extended the application of invasive VNS from epilepsy and depression to the treatment of obesity. Further research for non-invasive and invasive VNS is nevertheless required to understand their specific clinical benefits.

## Conclusion

The translation of non-invasive and invasive brain stimulation techniques from laboratory settings to patient care would profit from large clinical trials, such as ReCharge (Ikramuddin et al., 2014; Shikora et al., 2015) and VBLOC DM2 (Shikora et al., 2016). Comparable age and gender matched trials should combine brain stimulation techniques with standard treatment options, such as dieting, exercise or behavioral therapy. This combination may, on one hand, boost effects on appetite, eating behavior and weight loss. On the other hand, brain stimulation may sustain the effects of standard treatments that in most cases, are temporally limited (known as the yo-yo effect). Large clinical trials will also help to identify potential mediators of treatment effectiveness in different study samples that may in future help to develop more individualized therapy scenarios for obesity and its comorbidities.

## Author Contributions

BP searched the literature and wrote the manuscript.

## Conflict of Interest Statement

The author declares that the research was conducted in the absence of any commercial or financial relationships that could be construed as a potential conflict of interest.

## Supplementary Material

TABLE S1 Information of relevant tDCS, rTMS, VNS und DBS studies that were conducted to assess the stimulation technique’s efficiency to modulate food craving and food consumption.
