# Supplementary material for: Invasive and Non-invasive Stimulation of the Obese Human Brain
Source: Front Neurosci. 2018 Nov 29;12:884. doi: 10.3389/fnins.2018.00884 (PMC6281888; doi:10.3389/fnins.2018.00884)
Supplement: TABLE S1 — Information of relevant tDCS, rTMS, VNS und DBS studies that were conducted to assess the stimulation technique’s efficiency to modulate food craving and food consumption. [file Table_1.pdf]

First author et al.	Published in	Randomized	Sample size	Weight class (BMI, kg, lbs)	Gender (m/f)	Age (mean +/- standard deviation)	Method	single/repetitive session(s)	Target region(s)/side	Side of stimulation	Session length (tDCS)/stimuli per session (rTMS/DBS/VNS)	Control condition (sham)	Outcome (craving, liking, intake)	Effect duration	Effect size
Grundeis et al.	2017	yes	25	obese 102.5 kg ± 11.8 kg 82-130kg0 BMI 36.5 ± 4.1 31.4-45	f	28.8 ± 6.0 range 18-43	tDCS	single	Left dorsolateral prefrontal cortex (DLPFC)	cathode left DLPFC F8; anode over right frontal operculum AF7; vice versa	20 min; 2 mA	sham-controlled	no confimed effect on carving or calorie consumption		
Goldman et al.	2011	yes	19	82.02 kg ± 25.31 kg BMI: 27.25 ± 6.24 (26.3% overweight, 31.6% obese)	68.4% f	32.47 ± 10.85	tDCS	single	prefrontal cortex	anode right PFC (F4); cathode left PFC (F3)	20 min; 2 mA	sham-controlled	greater significant reduction of carving especially sweet food and carbohydrates; no difference food intake	temporary	d = .55
Fregini et al.	2008	yes	23		21 f	23.7 ± 7.2	tDCS	single 3 sessions (anodal, cathodal, sham)	dorsolateral prefrontal cortex (DLFPC)	anode left/cathode right; anode right/cathode left	20 min; 2 mA (constant current)	sham-controlled	anode right/cathode left: food craving reducted; less frequend fixed at food-related pictures; consumed less food; anode left/cathode right: craving levels did not increase; active stimulation: caloric ingestion significantly lower		
Kekic et al.	2014	yes	17	BMI 23.81 ± 2.60 range 19.85-29.28 70.6% healthy (18.5-24.9) 29.4% overweight (25-25.9)	f	19-55 26.41 ± 8.30	bilateral tDCS	single	dorsolateral prefrontal cortex (DLFPC)	anode right(F4)/cathode left(F3) DLPFC	20 min; 2 mA	sham-controlled	reducted: carving for sweet food not savoury; no difference: TD, food consumption	temporary	
Lapenta et al.	2014		9	BMI 21.9 ± 1.63 range 18.96-24.44 all normal weight	f	20-27 23.4 ± 2	bilateral tDCS	single	DLPFC	anode right/cathode left	20 min; 2 mA; one week interval between real and sham	sham-controlled	reducted frontal N2 component; enhanced P3a component of response to No-go stimuli (food or furniture) reduction caloric intake; food carving		
Montenegro et al.	2012	yes	9	overweight 84.7 kg (74.6-137.7kg) BMI 28.2 (25.2-43.5)	5 m; 4 f	20-32 24	tDCS	single	DLPFC	left (anode)	20 min; 2 mA	sham-controlled	left anodal: desire to eat decreased; greater effect tDCS + exercise: desire to eat & hunger		d = 3.0
Gluck et al.	2015	yes	9	obese 94 ± 15 kg	3m, 6f	42 ± 8	tDCS	repetitive 3 sessions of tDCS or sham over 9 days (total of 6 sessions over 3 years)	LDLPFC	anode F3 cathode above right eye	40 min; 2 mA	sham-controlled	consumed fewer kilocalories per day; significant fewer kilocalories from sode and fat; greater weight loss during anodal vs cathodal		
Jauch-Chara et al.	2014	yes	14	BMI 20-25 22.65 ± 0.34 (SEM)	m	21-28 24.81 ± 0.58 (SEM)	tDCS	repetitive	Right dorsolateral prefrontal cortex (DLPFC)	anode right DLPFC; cathode left supraorbital	2x 8 d daily stimulated tDCS/sham conditions 2-4 wk apart; 20 min; 1mA; fade in/out: 8s	sham-controlled	anodal: reductes caloric intake 14% (in comparison with sham) no effect: protein intake and fat		d > 1.09
Ljubisavljevic et al.	2016	yes	27	BMI 25.6 ± 4.4 77.3 ± 18.3 kg	19 m; 8 f	21.3 ± 2	tDCS	repetitive	right DLPFC	anode right/cathode left	5 days 1 session per day; 20 min; anode right/cathode left; 2 mA current density kept at 0.06 mA/cm2; 1min ramp-up/ramp-down	sham-controlled	single session: reducted intensity of current food carving; repetitiv session: reducted habitual experiences of food carving; decreased carving for fast food & sweets, lesser for fat; no significant effect on carving for carbohydrates; no assossiation with initial weight or weight	after 5-days; after 30-days: repetitive: current and habitual food carving significant reducted; single: no significant effect	
Barth et al.	2011	yes	10	mean: BMI 27,8 165.8lb SD: 8,0 50.3lb (healthy)	f	Mean: 28,3 SD: 6,5	single session rTMS	single	left prefrontal cortex (PFC)	left	10 Hz, 100% resting motor threshold, 10 s-on, 20 s-off for 15 min; 3000 pulses in a single 15 min session	sham-controlled	Cravings significantly lower regardless real or sham; not significant: -condition main effect and time by condition interaction -differance in hours since last ate		d = 0.09
Camus et al.	2009	no	Exp1 :56 Exp2: 15	Normal-weight BMI 18-25	1: m 30 2: m 8	1: mean 22 range 19-26 2: mean 21 range 19-25	rTMS; inibitory	single	Right dorsolateral prefrontal cortex (DLPFC) (sham: vertex)	right	Single 15 min, 50% of stimulator maximum 1 Hz rTMS train, 900 pulses	sham-controlled	1: -downmodulation of computation of goals values 2: effect might be specific to computation of goals values		
Uher et al.	2005	yes	28	BMI: real 27.7 ± 5.5 sham: 23.3 ± 5.3	f	real: 25.2 ± 5.4 sham: 26.4 ± 4.9	rTMS	single	Left dorsolateral prefrontal cortex	left	Single session, 10 Hz, intensity 110%; 1000 pulses over 20 min	sham-controlled	Inhibits the development of craving		d = .85
Lowe et al.	2008	?	28	BMI: mean 23.64 SD 3.352 normal weight	f	mean 20.43 SD 1.345	rTMS, cTBS	pre-/post-stimulation + active cTBS, sham cTBS	Left dorsolateral prefrontal cortex (dlPFC)	left	1 week intersession intensity 80% RMT; 40s continuous train; 600 pulses in theta burst pattern 3 stimuli at 50Hz repeated at 5Hz frequency	sham-controlled	caloric intake higher; high calorie food prefered over low calorie;		
Kim et al.	2008	yes	57 obese adults TMS:29 Sham: 28	BMI: -TMS: 86.90 ± 23.61 -Sham: 78.90± 17.04	TMS: m 13(44.8%) f 16(55.2%) Sham: m 13 (46.4%) f 15 (53.6%)	TMS: 38.66 ± 10.41 Sham: 43.32 ± 11.92	Repetitive transcranial mangnetic stimulation (rTMS)	repetitive 4 sessions	Left dorsolateral prefrontal cortex (DLPFC)	left	2 weeks intervention 2 weeks F/U; 4 sessions; 20 trains of 5 s with 55-s inter-train intervals; frequency 10 Hz; intensity 110% of individual´s motor threshold; 1000 pulses over 20 min	sham-controlled	reduction: weight, BMI, fat mass VAT kilocalories, protein, fat intake; increased: feeling of fullness; no change: hunger and desire	2 weeks	
Ruiz-Tovar et al.	2014	yes	105; group 1: 45; group 2: 45; group 3: 15	(group 1/2/3) BMI: 46.1±7.5/45.3±6/32.5±2.4 weight: 123.3±25.1/121.3±20.6/82.8±10.2 kg	80% f; 20% m	45.6 ± 14.3 (21-74)	percutaneous electrical neurostimulation(PENS)	12 sessions	dermatome T6		30 min session/week for 12 weeks; 20Hz (0-20 mA)	control group	group 1: significant reduction weight(7.1±1.5kg), BMI(2.7±0.5), appetite along with diet; 10.7±2.6 % EWL		
Ruiz-Tovar et al.	2016		150	obsese pat, BMI 30-40			PENS		dermatome T6				weight loss after 12 weeks: 11.8±2.6kg; 3 months after completing 14.6±2.7kg; 9 months 14.5±2.8kg; appetite (VAS) pre 6; after treatment 1; 3 months 1; 9 months 4; appetite is restored after 3 months, no weight regain	3 months	
Ikramuddin et al.	2014	yes	239 VNB: 162 sham: 77	BMI 41 ± 3 VNB: 113 ± 13kg sham: 116 ± 14kg comorbidity: (VNB/sham) DM2 9(6%)/6(8%); Hypertension 63(39%)/32(42%); Dyslipidemia 91(56%)/46(60%); OSA 33(20%)/23(30%)	VNB: 141 (87%) f sham: 62 (81%) f	VNB: 47 ± 10 Sham: 47 ± 9	invasive VNS	invasive 12 month period	anterior and posterior vagal nerve (gastroesophageal junction)		6-8 mA; 12h/d	sham-controlled	VNB: 24.4% excess weight loss (9.2% of initial body weight loss) sham: 15.9%(6.0%) mean difference 8.5%; VNB: 25% of pat. achieved 20% or more EWL, 38% achieved 25% or more loss	12 months	
Shikora et al.	2015	yes	239	BMI 41 ± 3 VNB: 113 ± 13kg sham: 116 ± 14kg comorbidity: (VNB/sham) DM2 9(6%)/6(8%); Hypertension 63(39%)/32(42%); Dyslipidemia 91(56%)/46(60%); OSA 33(20%)/23(30%)	VNB: 141 (87%) f sham: 62 (81%) f	VNB: 47 ± 10 Sham: 47 ± 9	invasive VNS	18 month result	anterior and posterior vagal nerve (gastroesophageal junction)		unblinding	sham-controlled	18-month weight loss vBloc: 23% EWL(8.8%TWL) sham: 10%EWL(3.8%TWL) vBloc: maintained 12-month weight loss 23%EWL (9.7%) sham: regained over 40% of the 17%EWL(6.4%TWL) by 18 month, most weight regain preceded unblinding	18 months	
Shikora et al.	2016		28	T2DM; obese pat.; 107 ± 16kg BMI 37 ± 3	17 f (61%)	51 ± 9	invasive VNS	2 year results	vagal nerve		5000 Hz; 3-8 mA for at least 12h each day (goal: 6mA for 14h/d	no control	57% achieved 20% EWL/5% TWL; 43% achieved 25%EWL/7.5%TWL; 30% achieved 10%TWL	2 years	
Whiting et al.	2013		3	(lbs/BMI) 278.7/49.4(HTN); 326/48.1(sleep apnoe, DM2;HTN; migraine); 314/45(lower-extremity edema)	2 f, 1 m	60, 50, 45	DBS	35 months	lateral hypothalamic area (LHA)	bilateral	monopolar or bipolar stimulation; 90 µsec pulse width; 185 Hz		significant weight loss 2 patients; 1 stable; increase RMR certain contacts/voltages		
Ho et al.	2015						DBS		lateral hypothalamic area (LHA)						
Harat et al.	2016		1	151.4kg	f	19	DBS		nucleus accumbens	bilateral	both electrodes bipolar stimulation; 208 µs pulse width, 130 Hz, 2 mA-3.75 mA		14 months after surgery 138kg, BMI 48.3; no feeling of increased appetite nor need for food; accidentals switch off of pacemaker: increased carving for food		
